# Understanding the blood brain barrier: current state and therapeutic implications

**DOI:** 10.1016/j.ebiom.2025.105704

**Published:** 2025-04-09

**Authors:** eBioMedicine

Why are some central nervous system (CNS) drugs effective in some individuals and for specific pathologies, when they might not work as well in others? Scientists have been puzzled by this question and the complexities of the blood–brain barrier (BBB) for over a century and are still exploring ways to transport drugs across this barrier to treat conditions in which the BBB is affected, such as ageing, neurodegenerative diseases, brain tumours, long COVID, epilepsy, stroke and traumatic brain injury.

The BBB serves as a protective, semipermeable, and highly regulated vascular interface, characterised by selective transporters and tight junctions between the endothelial cells that line blood vessels and the astrocytic endfeet that connect BBB endothelial cells to neurons, facilitating neurovascular communication.

A study published in Nature in February, 2025 led by Carolyn Bertozzi, a Nobel-prize winning chemist at Stanford University, identified brain endothelial glycocalyx dysregulation as a molecular mechanism that contributes to BBB dysfunction in ageing and neurodegenerative diseases, revealing new insights into how the BBB works. The glycocalyx layer is a complex network composed of glycans and diverse glycoconjugates (including proteoglycans, glycoproteins, and glycolipids) that coats the luminal surface of the BBB. This layer is essential for facilitating various cellular processes, such as signalling, adhesion, transport, and the maintenance of cell morphology. In this landmark study, the authors observed a downregulation in mucin-type O-glycosylation in brain endothelial cells as assessed in mouse ageing, human Alzheimer's disease, and Huntington's disease data. Further, the reduced brain endothelial mucin-type O-glycosylation resulted in increased BBB permeability and cerebral bleeding in mice. Notably, a brain endothelial cell-targeted adeno-associated virus injected retro-orbitally in the mice led to the overexpression of mucin-type O-glycan biosynthetic enzymes in brain endothelial cells. This intervention restored the integrity of the BBB, decreased neuroinflammation markers, and enhanced cognitive function in aged mice. These findings suggest that restoring the glycocalyx of brain endothelial cells could be a promising therapeutic strategy for addressing BBB disruption. However, it remains to be determined whether mucins play a more substantial role in transporting specific molecules or excluding others, as well as how this protein might facilitate drug transport across the BBB.

Genetic and environmental factors might also affect BBB integrity. The *APOE-ε4* genotype is one of the largest risk factors for Alzheimer's disease. A recent study published in eBioMedicine revealed mechanistic insights into how the *APOE* genotype and exercise affect brain endothelial cell barrier function and glucose metabolism. Using induced pluripotent stem cells homozygous for the *APOE-ε3* and *APOE-ε4* alleles, differentiated to resemble brain microvascular endothelial cells (BMECs), the authors showed that *APOE-ε4* BMEC exhibited impaired barrier function by reducing SIRT1, as well as decreasing BMEC glucose metabolism by impairing insulin signalling. Serum from exercise-trained individuals revealed genotype-dependent dimorphic effects on SIRT1; however, it did not alter BMEC barrier function or metabolism. The authors suggested that additional cues from exercise might be required to decrease Alzheimer's disease pathologies. A recent study published in Nature Neuroscience showed that loss of BBB integrity induced by stress-related inflammation in mice was prevented by neurovascular endocannabinoids, resulting in stress resilience. The authors reported an upregulation of astrocytic cannabinoid receptor gene (*Cnr1*) and cannabinoid receptor 1 in the nucleus accumbens (NAc) astrocytes of male mice resilient to chronic social defeat stress, as well as a decrease in *Cnr1* expression in postmortem NAc tissue from male donors with major depressive disorder. In the female resilient mice, *Cnr1* was upregulated in the prefrontal cortex but not in the NAc, highlighting the need to study the sex-specific effects of stress on the BBB and the importance of astrocytic *Cnr1* as a therapeutic target for restoring BBB integrity. Therefore, based on the rodent model data, the BBB phenotype appears to be influenced by factors such as age, sex, environment, and disease pathology, which might somewhat explain the variability in responses to CNS drugs in different individuals and pathologies.

Due to the restrictive nature of the BBB, delivering drugs across this barrier presents a substantial challenge. Some examples of the ongoing efforts to develop methods for overcoming the BBB include engineering of tissue-sensing T cells, ultrasound-based methods, nanotherapeutics (which can penetrate the BBB), and engineered commensal microbes and intracalvariosseous (ICO) approaches (which can bypass the BBB).

For example, T cells that can cross the BBB are being engineered to express synthetic Notch receptor proteins, which only bind to a brain specific protein. This targeted interaction activates genes within the T cells, enabling them to produce the desired therapeutic agent directly in the brain while sparing other tissues. Ultrasound-based methods to deliver drugs across the BBB do so by facilitating the opening of endothelial junctions around the brain capillaries, allowing the concomitantly administered drug to penetrate the BBB, using sound waves to resonate with microbubbles co-administered intravenously with a drug. To ensure the consistency and safety of this technology, quality assurance strategies are essential. In a recently published study in eBioMedicine, the authors introduced a quality assurance protocol based on passive acoustic detection for the focused ultrasound FUS-induced BBB opening procedure. The authors showed that the FUS device met quality assurance standards in 9 of 10 patients with glioma. However, 4 of the 9 cases initially failed the acoustic coupling quality assurance. The acoustic coupling procedure was repeated until quality assurance was successfully achieved in 3 of those 4 cases, indicating that the proposed protocol could be effectively integrated into a clinical FUS system. FUS technology has previously been used in clinical trials to deliver monoclonal antibodies for Alzheimer's disease, with ongoing trials such as NCT05469009. Additionally, there are both completed and ongoing trials investigating the use of FUS in patients with Parkinson's disease (NCT05565443).

Nanotherapeutics involves using lipid-based and polymer-based nanoparticles to encapsulate, transport, and deliver drugs and nucleic acids to the brain. A Phase 0 clinical trial NCT03020017 involving human patients with glioblastoma demonstrated a proof of concept that siRNA nanostructures could cross the BBB to reduce the expression of target proteins. A recent review published in eBioMedicine examined the role of extracellular vesicles—lipid-enclosed nanovesicles—in drug delivery. The authors proposed that engineering extracellular vesicles with optimised brain-targeting capabilities, combined with precisely selected therapeutic cargos, represents a promising strategy to enhance extracellular vesicle-based therapies.

In a March, 2025 study published in Cell, the authors engineered an intranasal strain of *Lactobacillus plantarum* that specifically targets the release of therapeutic payloads in the mouse olfactory epithelium, facilitating transport to the brain while bypassing the BBB. The authors administered this engineered *L plantarum*, designed to secrete three appetite regulating hormones (leptin, alpha-melanocyte-stimulating hormone [α-MSH], and brain-derived neurotrophic factor) to mice on a high-fat diet that significantly reduced their body weight gain, reduced the fat mass deposition, and improved glucose metabolism. Currently, glucagon-like peptide receptor agonists (GLP-1 RA)s are being used for diabetes and obesity and being investigated for their repurposing potential in cognitive and mental disorders. The potential for GLP-1 RAs to be administered intranasally is exciting, as this route of administration bypasses first-pass metabolism in the liver, thereby reducing systemic side effects.

A study published in eBioMedicine explored the use of natural microchannels between the skull's bone marrow and the dura mater as a method for drug delivery in stroke treatment in mice. The authors showed that ICO injection allows for the delivery of drugs to the brain parenchyma through these microchannels, bypassing the BBB and offering greater delivery efficiency compared with intravenous injection. The ICO injection was found to be safe and feasible, as it did not lead to skull infections or compromise the BBB. Additionally, drug accumulation in the brain increased following ICO injection, which contributed to nerve repair, reduced neuronal apoptosis, and lowered the expression of inflammatory factors.

Finally, there are other strategies that have been developed to cross the BBB. A comprehensive discussion of these strategies can be found in a personal view article by Pedder and colleagues published in *The Lancet Neurology*. For instance, cerebrospinal fluid delivery and intracranial delivery are direct yet invasive methods that have yet to demonstrate efficacy in clinical trials. Techniques that use membrane transporters and receptor-mediated transcytosis are generally less invasive, but they might lead to off-target effects. The authors emphasise that successful advancements in drug delivery across the BBB will rely on robust preclinical animal research and optimally designed and adequately powered clinical trials, that consider treatment timing, demographics, and genetic factors in patients with BBB disruption.

Understanding the BBB will be the key to identifying therapeutic targets and developing drug delivery methods that can reach those targets in pathologies that disrupt the BBB. At eBioMedicine, we encourage studies that advance BBB research using human, animal, or organoid models, as well as those leveraging large-scale MRI datasets and innovative machine learning approaches.

